# The endoscopic endonasal approach is not superior to the microscopic transcranial approach for anterior skull base meningiomas—a meta-analysis

**DOI:** 10.1007/s00701-017-3390-y

**Published:** 2017-11-10

**Authors:** Ivo S. Muskens, Vanessa Briceno, Tom L. Ouwehand, Joseph P. Castlen, William B. Gormley, Linda S. Aglio, Amir H. Zamanipoor Najafabadi, Wouter R. van Furth, Timothy R. Smith, Rania A. Mekary, Marike L. D. Broekman

**Affiliations:** 1 Brain Center Rudolf Magnus, Utrecht University Medical Center, Utrecht, The Netherlands; 2 Department of Neurosurgery, University Medical Center Utrecht, HP G03.124, PO Box 85500, 3508GA Utrecht, The Netherlands; 3 Cushing Neurosurgery Outcomes Center, Department of Neurosurgery Brigham and Women’s Hospital, Harvard Medical School, Boston, MA USA; 4 School of Pharmacy, Department of Pharmaceutical Business and Administrative Sciences, MCPHS University, Boston, MA USA; 5 Department of Anesthesiology, Brigham & Women’s Hospital, Harvard Medical School, Boston, MA USA; 6 Department of Neurosurgery, Leiden University Medical Center, Leiden, The Netherlands; 7 Department of Neurology, Massachusetts General Hospital, Harvard Medical School, Boston, MA USA

**Keywords:** Endoscopic transsphenoidal surgery, Microscopic transcranial surgery, Tuberculum sellae meningioma, Olfactory groove meningioma, Gross total resection, Complications, Meta-analysis

## Abstract

**Object:**

In the past decade, the endonasal transsphenoidal approach (eTSA) has become an alternative to the microsurgical transcranial approach (mTCA) for tuberculum sellae meningiomas (TSMs) and olfactory groove meningiomas (OGMs). The aim of this meta-analysis was to evaluate which approach offered the best surgical outcomes.

**Methods:**

A systematic review of the literature from 2004 and meta-analysis were conducted in accordance with the PRISMA guidelines. Pooled incidence was calculated for gross total resection (GTR), visual improvement, cerebrospinal fluid (CSF) leak, intraoperative arterial injury, and mortality, comparing eTSA and mTCA, with p-interaction values.

**Results:**

Of 1684 studies, 64 case series were included in the meta-analysis. Using the fixed-effects model, the GTR rate was significantly higher among mTCA patients for OGM (eTSA: 70.9% vs. mTCA: 88.5%, p-interaction < 0.01), but not significantly higher for TSM (eTSA: 83.0% vs. mTCA: 85.8%, p-interaction = 0.34). Despite considerable heterogeneity, visual improvement was higher for eTSA than mTCA for TSM (p-interaction < 0.01), but not for OGM (p-interaction = 0.33). CSF leak was significantly higher among eTSA patients for both OGM (eTSA: 25.1% vs. mTCA: 10.5%, p-interaction < 0.01) and TSM (eTSA: 19.3%, vs. mTCA: 5.81%, p-interaction < 0.01). Intraoperative arterial injury was higher among eTSA (4.89%) than mTCA patients (1.86%) for TSM (p-interaction = 0.03), but not for OGM resection (p-interaction = 0.10). Mortality was not significantly different between eTSA and mTCA patients for both TSM (p-interaction = 0.14) and OGM resection (p-interaction = 0.88). Random-effect models yielded similar results.

**Conclusion:**

In this meta-analysis, eTSA was not shown to be superior to mTCA for resection of both OGMs and TSMs.

**Electronic supplementary material:**

The online version of this article (10.1007/s00701-017-3390-y) contains supplementary material, which is available to authorized users.

## Introduction

The mainstay of treatment for tuberculum sellae meningiomas (TSMs) and olfactory groove meningiomas (OGMs) is surgery. Goals of surgery include obtaining tissue for histopathological diagnosis and relieving pressure caused by the tumor on neighboring structures such as the olfactory nerves, anterior cerebral arteries, optic nerves, and pituitary gland. At the same time, these structures are very susceptible to manipulation, and damage to these structures can lead to great morbidity [51].

Traditionally, TSMs and OGMs are resected using a microscopic transcranial approach (mTCA). Various approaches have been described, including interhemispheric, pterional, bifrontal, and subfrontal mTCA [1, 2, 5–7, 9, 47, 51, 56, 64, 70]. In the last decade, however, as a result of the evolution of endoscopic surgery for pituitary adenomas, these meningiomas have been increasingly resected using an endonasal endoscopic transsphenoidal approach (eTSA), as first described by Jho et al. in 2004 [38]. Although the endoscopic approach is generally viewed as less invasive, with some studies suggesting that eTSA caused fewer postoperative changes on magnetic resonance imaging (MRI) compared to mTCA possibly indicating less manipulation [22], it has been suggested that eTSA results in higher rates of CSF leaks and potentially different outcomes (e.g., less GTR) [18, 42]. However, a direct comparison between eTSA and mTCA is currently lacking. Therefore, the aim of this systematic review and meta-analysis was to evaluate which approach (eTSA vs. mTCA) offers the best surgical outcomes.

### Search strategy and paper selection

To identify studies reporting on outcomes of surgically treated TSMs and OGMs, a systematic review of the literature was conducted in accordance with the Preferred Reporting Items for Systematic Reviews and Meta-Analyses (PRISMA) Statement [54]. Both PubMed and Embase databases were searched on September 12, 2016. Because the outcomes of endoscopic surgery were first described in 2004 and microscopic resection has seen a continuous improvement, only articles published in 2004 or later were included [26, 38]. The search strategy was drawn up using the keywords “meningioma,” “tuberculum sellae,” “olfactory groove,” and synonyms (Supplementary Table 1). Duplicates were removed using Endnote X7.5.

Two authors (IM and TO) independently screened the titles and abstracts of the articles for papers reporting surgical outcomes of resected OGMs and TSMs. After full-text screening, articles that reported outcomes of surgically treated OGMs and TSMs were included. Case reports, commentaries, congress abstracts, reviews, animal studies, studies describing an endoscopically assisted approach, studies reporting on the use of a keyhole approach, studies in pediatric patients (< 18 years old), re-operations, and cadaveric studies were excluded. Only literature in English and Dutch was reviewed. Discrepancies in selection were sorted out by discussion, and a senior author (MB) was consulted if the discrepancy could not be solved by discussion.

### Data extraction

The following study characteristics were extracted from the full text of the selected studies: study design, number of patients, follow-up duration, study geographic location, percentage of WHO II and III meningiomas, percentage of males in the study population, mean age of the study population, and surgery type (transcranial or endoscopic endonasal). The following outcomes were extracted: number of patients with GTR (defined as Simpson grade I or II), number of patients with preoperative visual problems, number of patients with improved vision post-surgery, postoperative cerebrospinal fluid (CSF) leakage, number of intraoperative arterial injury, and all-cause mortality (within 30 days after resection). Furthermore, perioperative blood loss, hospital length of stay, and operation length were extracted. Study quality was assessed with the adjusted Newcastle Ottawa Scale (NOS) [80]. If the study in question was a case series, comparability was ignored.

### Meta-analysis

Comprehensive meta-analysis (CMA) version 3 was used to calculate the separate overall incidence using the fixed-effect model with the inverse variance method and the random-effect model according to the method of DerSimonian and Laird [27] in the endonasal endoscopic and transcranial approach for the following variables: GTR, arterial injury, visual improvement, CSF leakage, and mortality. A resulting p-interaction value from the subgroup analysis comparing eTSA and mTCA was considered significant if <0.05. Study heterogeneity was assessed by calculating I-squared and *P*-values from the Cochrane Q test. Publication bias was assessed with Begg’s tests and was corrected for by a trim-and-fill method. Finally, a meta-regression was conducted on each of age, gender (dichotomized by male percentage below/above the median category), and continent (North America as the reference) for eTSA and mTCA separately. For visual outcomes, only continent could be assessed as a source of heterogeneity as not all patients presented with visual problems and baseline characteristics from this subgroup were not available. A subgroup analysis for tumor size and grade was not possible because of great variance in reporting.

## Results

**Fig. 1:**
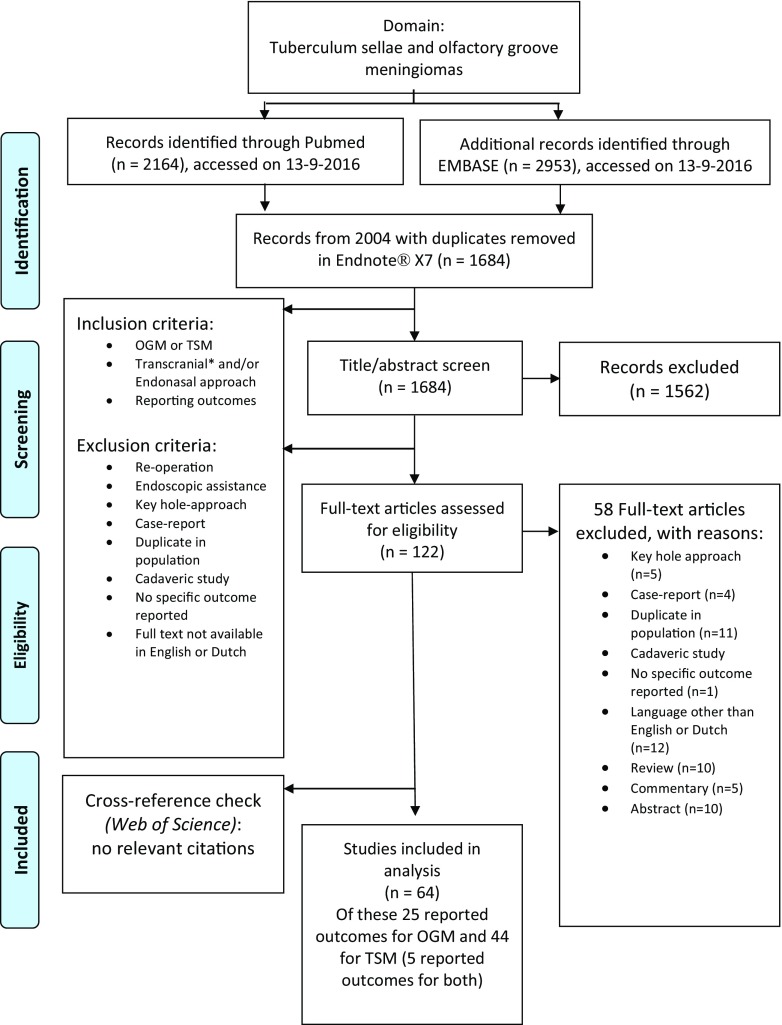
Flowchart. Abreviations: OGM: olfactory groove meningioma, TSM: tuberculum sellae meningioma

After removing duplicates, 1684 articles were identified. After screening for titles and abstracts, 1426 articles were excluded and 216 full texts were reviewed (Fig. 1). For TSM, 44 case series (of which 11 were in eTSA, 29 in mTCA, and 4 in both) were included in the meta-analysis for the different outcomes, including a total of 1444 patients [3, 5, 8, 11–13, 15, 16, 20, 21, 23, 25, 29, 30, 32, 34–36, 40, 41, 43, 45, 47–53, 56, 58, 61–63, 65, 66, 68, 69, 72, 73, 77, 79, 81, 82]. As for OGM, 25 case series (of which 6 were in eTSA, 18 in mTCA, and 1 in both) were included describing outcomes in 891 patients [2, 4, 6, 7, 17, 19, 22, 24, 25, 35, 37, 40, 44, 47, 55, 57, 60, 62, 64, 67, 68, 70, 75, 76, 78].

The median number of patients per study was 24 for TSM (Table 1) and 29 for OGM (Table 2). The average percentage of male patients was 27% for TSM and 32% for OGM. The median age was 51.0 for TSM and 52.0 for OGM. The median follow-up time was 6.0 years based on 35 studies for TSM [3, 5, 8, 12, 13, 15, 16, 21, 25, 29, 30, 32, 34, 36, 43, 45, 47–53, 56, 61, 62, 65, 66, 68, 72, 73, 77, 79, 81, 82] and 7.0 years based on 20 studies for OGM [2, 4, 6, 7, 17, 19, 22, 24, 25, 37, 44, 47, 55, 57, 60, 62, 67, 68, 76, 78]. The modified NOS score varied between three and four of seven among the TSM and OGM case series [3, 5, 8, 11–13, 15, 16, 20, 21, 23, 25, 29, 30, 32, 34–36, 40, 41, 43, 45, 47–53, 56, 58, 61–63, 65, 66, 68, 69, 72, 73, 77, 79, 81, 82]. Outcomes of the meta-analysis for TSM (Table 3) and OGM (Table 4) are shown.

**Table 1:** Study characteristics of tuberculum sellae menigoma (TSM) studies

Authors	TSM (N)	Mean age (range)	Meningioma grade: WHO II and WHO III (N)	% Male	Meningioma size	Approach	Mean follow-up (years)	Modified NOS*
Ali et al. [3]	30	48 (34–63)	0 and 0	43	NR	mTCA	2.5 (range: 0.5–4)	3
Bassiouni et al. [5]	62	53 (29–81)	NS	26	NR	mTCA	6 (range: 1.5–14)	3
Bohman et al. [9]	5	53 (24–77)	NS	40	Mean DM: 4.74 cm	eTSA	0.65 (range: 0.18–1.42)	4
Bowers et al. [11]	27	54 (23–77)	NS	18.5	NR	mTCA + eTSA	NR	3
Ceylan et al. [12]	23	52.9 (23–77)	NS	18.5	Mean DM 2.55 cm	eTSA	1.82 (range: 0.17–2.42)	3
Chen et al. [13]	6	49.8 (4–78)	NS	33	NR	mTCA	2.44 (range: 0.5–4.04)	4
Chokyu et al. [15]	34	55.7 (23–78)	0 and 0	15	Mean DM: 2.43 cm	mTCA	7.98 (range: 1.25–16.2)	3
Chowdhury et al. [16]	6	39.5 (29–52)	NS	33	Mean DM: 3.5 cm	eTSA	0.58 (range: 0.16–1)	4
Cook et al. [20]	3	40.3 (32–55)	NS	0	NR	eTSA	NR	3
Curey et al. [21]	20	59.1 (SD: 11.1)	0 and 0	15	Mean DM: 3.25 (SD: 1.38 cm)	mTCA	4.69 (SD: 2.83)	4
De divitiis et al. [23]	51	NS	NS	20	DM: 6: < 2 cm, 33: 2–4 cm, 5: > 4 cm	mTCA + eTSA	Range: 0.75–21	4
Della puppa et al. [25]	23	NS	NS	0	NR	mTCA	3.42 (range: 0.25–6.42)	3
Fatemi et al. [29]	23	40 (SD: 22)	NS	30	Mean DM: 3.08 cm	mTCA + eTSA	eTSA: 1.67 (range: 0.25–5), mTCA: 1.17 (range: 0.92–1.5)	4
Gadgil et al. [30]	5	51 (31–66)	0 and 0	40	Mean volume: 6.3 cm^3^	eTSA	1.25 (range: 0.25–2.25)	4
Ganna et al. [32]	24	53.8 (33–80)	0 and 0	17	Mean DM: 2.63 cm	mTCA	4.33 (range: 1.5–7.67)	3
Goel et al. [34]	85	NS	NS	NS	NR	mTCA	4 (range 0.5–9)	4
Hayhurst et al. [35]	9	48.7 (29–65)	0 and 0	42	NR	eTSA	Median follow-up 38.6 (range 12–60 months)	4
Jang et al. [35]	24	49.5 (25–70)	NS	21	Mean DM: 2.06 cm	mTCA	1.73 (range: 0.25–4.5)	3
Khan et al. [40]	20	56.5 (31–81)	0 and 0	30	Mean volume: 11.98 cm^3^	eTSA	NS	3
Kitano et al. [41]	28	Median: 55 (range:42–76)	NS	14%	Mean volume; 8.1 mm3 (range 0.7–31.4 mm3)	mTCA + eTSA	NS	3
Koutourousiou et al. [43]	70	57.3 (36–88)	0 and 0	16	Mean DM: 2.3 cm	eTSA	2.42 (range: 0.083–8.17	3
Landeiro et al. [45]	23	56.2 (38–77)	NS	35	NR	mTCA	2.6 (range: 0.5–10.3)	3
Leveque et al. [47]	18	63.8 (31–88)	NS		DM < 4.0 cm: 11, > 4.0 cm: 7	mTCA	4.74 (SD: 2.74)	4
Li et al. [48]	43	53.8 (24–68)	NS	28	DM: < 2 cm: 8, 2–4 cm: 22, > 4 cm: 13	mTCA	5.4 (range: 2–10)	3
Li-hua et al. [49]	67	48.7 (28–76)	NS	42	DM: < 3 cm: 29, > 3 cm: 38	mTCA	2.44 (range: 0.5–4.04)	4
Liu et al. [50]	19	NS	NS		NR	mTCA	1.24 (range: 0.33–3.83)	4
Mahmoud et al. [51]	58	56 (13–80)	NS	31	Mean DM: 2.9	mTCA	1.92 (up to 12 years)	4
Margalit et al. [52]	51	57.1 (28–83)	NS	32	Mean max DM 2.94 cm (SD: 1.07)	mTCA	3.51 (range 0.17–7)	3
Mathiesen et al. [53]	29	58.3 (30–84)	0 and 0	21	Mean max DM: 23.9 cm	mTCA	6 (1.5–10)	4
Nakamura et al. [56]	72	54.3 (30–86)	1 and 0	24	Mean max 2.5 cm	mTCA	3.8 (range: 0.33–19.8)	3
Nanda et al. [58]	24	NS	NS	NS	DM: < 3 cm: 3, 3–5 cm: 6, > 5 cm: 21	mTCA	Median: 1.5	4
Ogawa et al. [61]	29	58.9 (43–79)	2 and 0	26	NR	eTSA	2.98 (range: 0.5–4.92)	3
Padhye et al. [62]	3	66 (65–66)	0 and 0	0	Mean volume 25.7 cm^3^	eTSA	1.83 (range: 0.25–6)	4
Palani et al. [63]	41	NR	NR	37	NR	mTCA	Range: 0.5–4	4
Pamir et al. [65]	42	53 (24–79)	3 and 1	33	Range 7.5–210 mm^3^	mTCA	3.13 (range: 0.25 0 16)	3
Park et al. [66]	21	51	NS	14	Mean volume: 12.4 cm^3^	mTCA	6.33 (range: 1–12.6)	4
Refaat et al. [68]	16	NS	NS	19	Mean DM: 2.5 cm	mTCA	1.17 (range: 0.67–1.5)	3
Romani et al. [69]	52	Median: 59 (14–87)	1 and 0	19	Mean DM: 3.1 cm	mTCA	Median: 4.91 (range: 0.08–11.1)	3
Schick et al. [72]	53	52.6 (27–78)	NS	25	Mean DM 2.6 cm	mTCA	2.49 (range: 0.5–9)	4
Seol et al. [73]	86	49 (24–75)	NS	23	Mean Dm: 2.41	mTCA	3.25 (range: 0.6–12.2)	3
Terasaka et al. [77]	9	64 (57–83)	0 and 0	11	NR	mTCA	2.1 (0.5–5.92)	4
Wang et al. [79]	12	56.7 (40–67)	0 and 0	33	Mean DM: 3.03 cm	eTSA	2.1 (range: 0.5–5)	3
Wilk et al. [81]	18	50.5 (30–73)	0 and 0	17	Mean volume 6.915 mm^3^	mTCA	1.96 (range: 0.5–3.25)	4
Zhou et al. [82]	56	42.5 (21–69)	NS	46	DM: < 3 cm: 24, 3–5 cm: 26 > 5 m: 6	mTCA	2.29 (range: 0.08–3)	4

WHO, World Health Organization; SD, standard deviation; NR, not reported; DM, diameter; NS, not specified; mTCA, microscopic transsphenoidal approach; eTSA, endoscopic transsphenoidal approach; NOS, Newcastle Ottawa Scale

*The modified NOS score varied between 3 and 4; the difference was mainly caused by variation in specifying completeness of follow-up

**Table 2:** Study characteristics of olfactory groove meningioma (OGM) studies

Authors	OGM (N)	Mean age (range)	Meningioma grade: WHO II and WHO III (N)	% Male	Meningioma size	Mean follow-up in years (range)	Approach	Modified NOS*
Aguiar et al. [2]	21	50 (21–76)	NR	29	Mean DM: 4.3 (SD: 1.1 cm)	4.17 (0.25–10)	mTCA	3
Banu et al. [4]	6	61.4 (41–77)	NR	26	Mean volume 19.6 cm^3^	1.54 (0.083–7)	eTSA	3
Bassiouni et al. [6]	62	51 (NS)	1 and 0	27	Mean DM: 5.2 cm (SD: NS)	5.6 (1–13)	mTCA	4
Bitter et al. [7]	61	60 (NS)	3 and 2	34	< 2 cm: 5%, 2–4 cm: 6.5%, > 4 cm: 88.5%	9.33 (0.67–19.9)	mTCA	3
Ciurea et al. [17]	59	52.9 (20–76)	3 and 0	41	2–4 cm: 16, 4–6: 32, > 6: 11	7 (0.75–12)	mTCA	3
Colli et al. [19]	17	53.12 (19–76)	0 and 0	6	NR	4.25 (0.083–17.4)	mTCA	4
De almeida et al. [22]	20	eTSA: 53.1 (NS), mTCA: 49.7 (NS)	NR	eTSA: 20, mTCA: 20	Volume: eTSA: 35.7 cm3, mTCA: 36.2 cm3	4.08 (0.24–9.58)	eTSA + mTCA	5†
De divitiis et al. [24]	4	49.25 (35–65)	0 and 0	25	Mean DM: 4.0 cm (SD: NR)	0.81 (0.75–1)	eTSA	3
Della Puppa et al. [25]	20	NS	NR	NR	DM: < 3.5 CM	3.42 (0.25–6.42)	mTCA	4
Hayhurst et al. [35]	8	50.2 (30–76)	0 and 0	11	NR	Median: 3.22 (1–5)	eTSA	4
Jang et al. [37]	40	59.1 (33–74)	7 and 1	58	Mean DM: 4.59 cm (SD: NS)	4.86 (0.25–15.33)	mTCA	3
Khan et al. [40]	11	NS	0 and 1	ns	NR	NS	eTSA	4
Koutourousiou et al. [44]	45	57.1 (27–88)	1 and 0	36	Mean DM: 4.41 cm (SD: NR)	2.71 (0.25–9.58)	eTSA	3
Leveque et al. [47]	34	NS	NR	NR	NR	4.74 (0.5–10)	mTCA	
Mukherjee et al. [55]	33	41 (4–89)	12 and 0	33	NR	3.17 (0.5–5.17)	mTCA	4
Nakamura et al. [57]	82	57.8 (33–91)	NR	23	Mean DM: 4.5 cm (SD: NR)	5.28 (0.33–22.5)	mTCA	3
Nanda et al. [60]	57	NS	NR	40	Mean DM: 4.41 cm (SD: NR)	1.18 (1–1.25)	mTCA	3
Padhye et al. [62]	8	52 (28–74)	0 and 0	25	Mean volume: 25.7 cm^3^	1.83 (0.25–6)	eTSA	3
Pallini et al. [64]	113	57 (17–82)	NR	35	Mean DM: 5.4 cm	Median 7.42 (0.167–27)	mTCA	3
Pepper et al. [67]	19	51 (15–68)	1 and 3	53	NR	3. 42 (NR)	mTCA	3
Refaat et al. [68]	14	50.8 (35–67)	NR	21	Mean DM: 5.8 cm (SD: NR)	1.17 (0.75–1.5)	mTCA	3
Romani et al. [70]	66	57 (38–85)	8 and 0	47	Mean DM: 4.7 cm (SD: NR)	Median: 4.92 (0.083–11.1)	mTCA	4
Slavik et al. [75]	29	54 (36–68)	NR	41	NR	NR	mTCA	3
Spektor et al. [76]	80	55 (16–85)	2 and 0	28	Mean DM: 4.6 cm (SD: NR)	5.9 (0.5–13.7)	mTCA	3
Tuna et al. [78]	25	NS	NR	NR	NR	4.87 (1.17–9.33)	mTCA	4

NS, Not specified; NR, not reported; DM, diameter; eTSA, endoscopic transsphenoidal approach; mTCA, microscopic transcranial approach; SD, standard deviation; NOS, Newcastle Ottawa Scale

*The modified NOS score varied between 3 and 4; the difference was mainly caused by not specifying the completeness of follow-up

†One OGM study (13) compared eTSA to mTCA and was given 5 stars

**Table 3:** Outcomes of the *tuberculum sellae meningioma* (TSM) meta-analysis

Outcomes in TSM	No. of studies	Prevalence % (95% CI) fixed and random	P-Interaction fixed and random effects	I^2^ (%)	Cochrance Q test (*P*-value)	Begg’s test (P-value) for publication bias	Meta-regression on age	Meta- regression on gender, (<27% vs. ≥27% males)	Meta- regression on continent (North America as reference)
GTR							Coefficient (P-value); random effect		Overall P-value; random effect
eTSA; fixed	14	83.0 (76.7–88.0)	0.34						
Random		83.1 (76.2–88.3)	0.33	0.00	0.74		0.05 (0.26)	0.28 (0.50)	0.62
						0.31			
MTCA; fixed	31	85.8 (83.6–87.9)							
Random		86.1 (83.5–88.4)		28.4	0.07		0.01 (0.78)	**0.49 (0.03)**	**0.02**
Visual improvement									
eTSA; fixed	12	77.7 (70.3–83.7)	**<0.01**						
Random		77.0 (64.8–85.9)	**0.04**	7.90	0.37		*	*	0.42
						0.14			
MTCA; fixed	28	60.7 (57.3–64.0)							
Random		62.6 (55.2–69.3)		77.4	**< 0.01**		*	*	0.30
CSF Leak									
eTSA; fixed	15	19.3 (14.1–25.8)	**< 0.01**						
Random		19.3 (14.1–25.8)	**< 0.01**	0.00	0.50		0.01 (0.77)	0.27 (0.51)	0.16
						0.98			
MTCA; fixed	24	5.81 (4.33–7.75)							
Random		5.81 (4.33–7.75)		0.00	0.93		0.03 (0.52)	0.02 (0.96)	0.94
Arterial injury									
eTSA; fixed	12	4.89 (2.33–9.94)	**0.03**						
Random		4.89 (2.33–9.94)	**0.03**	0.00	0.97		−0.04 (0.54)	−0.51 (0.52)	0.69
						**< 0.01**†			
MTCA; fixed	27	1.86 (1.13–3.05)							
Random		1.86 (1.13–3.05)		0.00	0.99		−0.01 (0.96)	−0.14 (0.79)	0.78
Mortality									
eTSA; fixed	10	5.15 (2.39–10.8)	0.14						
Random		5.15 (2.39–10.8)	0.14	0.00	0.85		−0.02 (0.81)	0.00 (0.99)	0.91
						**< 0.01**‡			
MTCA; fixed	30	2.67 (1.77–4.02)							
Random		2.67 (1.77–4.02)		0.00	0.99		−0.02 (0.76)	−0.34 (0.43)	0.99

GTR, Gross total resection; mTCA, microscopic transcranial approach; eTSA, endoscopic transsphenoidal approach; CSF, cerebrospinal fluid

*Meta-regression for age and gender was not possible for visual outcomes because the numbers were given for all subjects in the study and not all patients presented with visual problems

†Egger’s p-value for publication bias was 0.35, non-significant

‡Egger’s p-value for publication bias was 0.45, non-significant

**Table 4:** Outcomes of the olfactory groove meningioma (OGM) meta-analysis

Outcomes in OGM	No. of studies	Fixed and random prevalence % (95% CI)	P-Interaction fixed and random effects	I^2^ (%)	Cochrance Q test (P-value)	Begg’s test (P-value) for publication bias	Meta-regression on age	Meta-regression on gender, (< 29% vs. ≥ 29% males)	Meta-regression on continent (North America as reference)
GTR							Coefficient (P-value); random effect		Overall *P*-value; random effect
eTSA; fixed	7	70.9 (60.3–79.7)	**< 0.01**						
Random		72.9 (59.4–83.2)	**< 0.01**	0.00	0.45		**−0.18 (0.05)**	0.52 (0.44)	0.15
						0.48			
MTCA; fixed	18	88.5 (85.9–90.7)							
Random		89.1 (85.8–91.8)		36.5	0.06		0.05 (0.17)	0.11 (0.82)	0.30
Visual improvement									
eTSA; fixed	4	64.5 (37.9–84.4)	0.33						
Random		63.3 (30.9–87.0)	0.40	65.5	0.03		*	*	0.34
						0.25			
MTCA; fixed	9	50.6 (42.9–58.4)							
Random		47.4 (31.9–63.5)		68.6	**< 0.01**		*	*	0.57
CSF leak									
eTSA; fixed	7	25.1 (17.5–34.8)	**< 0.01**						
Random		20.1 (10.4–35.1)	**0.04**	25.8	0.22		0.01 (0.94)	−0.30 (0.60)	0.54
						0.30			
MTCA; fixed	17	10.5 (8.22–13.4)							
Random		9.11 (6.01–13.6)		60.2	**< 0.01**		**−0.12 (< 0.01)**	0.07 (0.91)	0.22
Arterial injury									
eTSA; fixed	7	3.88 (1.55–9.43)	0.12						
Random		3.89 (1.55–9.43)	0.12	0.00	0.98		−0.06 (0.67)	0.97 (0.38)	0.79
						**< 0.01**†			
MTCA; fixed	17	1.62 (0.87–2.98)							
Random		1.62 (0.87–2.98)		0.00	0.99		−0.10 (0.22)	0.22 (0.81)	0.87
Mortality									
eTSA; fixed	7	4.27 (1.50–11.6)	0.88						
Random		4.27 (1.50–11.6)	0.88	0.00	0.94		−0.06 (0.68)	1.20 (0.34)	0.78
						0.21			
MTCA; fixed	19	3.92 (2.66–5.75)							
Random		3.92 (2.66–5.75)		0.00	0.74		−0.04 (0.44)	**1.02 (0.02)**	0.08

GTR, gross total resection; mTCA, microscopic transcranial approach; eTSA, endoscopic transsphenoidal approach; CSF, cerebrospinal fluid

*Meta-regression for age and gender was not possible for visual outcomes because the numbers were given for all subjects in the study and not all patients presented with visual problems

†Egger’s p-value for publication bias was 0.50, non-significant

## Gross total resection

For TSM, GTR after eTSA was reported in 14 studies [8, 11–13, 16, 20, 23, 29, 30, 40, 43, 61, 62, 79] and after mTCA was reported in 31 studies [3, 5, 11, 13, 15, 21, 23, 25, 29, 32, 34, 36, 45, 47–49, 51–53, 56, 58, 63, 65, 66, 68, 69, 72, 77, 79, 81, 82]. In a fixed effect model, the overall incidence for GTR was not significantly different comparing eTSA (incidence = 83.0%; 95% CI = 76.7–88.0%, p-heterogeneity = 0.74, I^2^ = 0%, 221 patients) to mTCA (incidence = 85.8% (95% CI = 83.6–87.9%, p-heterogeneity = 0.07, I^2^: 28.4%, 1223 patients) (p-interaction value = 0.34). In meta-regression, TSM studies with lower percentage of males had a higher rate of GTR (*p* = 0.03). Studies conducted in Europe and Africa had significantly higher rates of GTR than those in North America (*p* = 0.02). Begg’s test for publication bias was non-significant (*p* = 0.31) (Table 3).

**Fig. 2:**
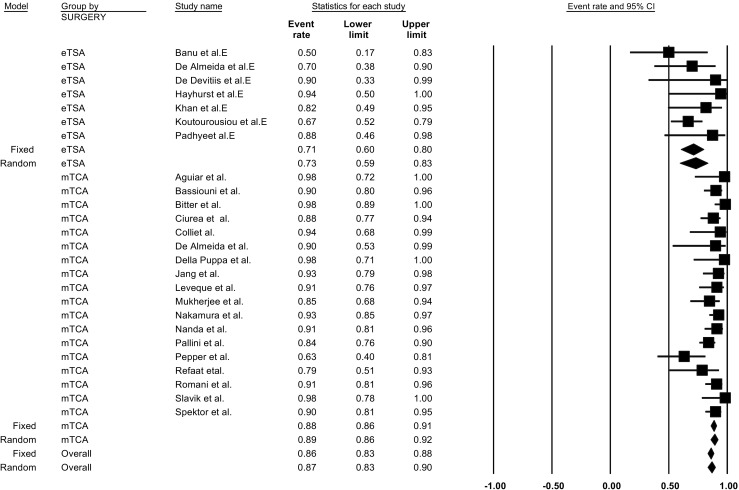
Pooled prevalence of gross total resection by approach for olfactory groove meningioma resection: endoscopic transsphenoidal approach vs. microscopic transcranial approach. P-interaction value < 0.01. eTSA, endoscopic transsphenoidal approach; mTCA, microscopic transcranial approach

For OGM, GTR was specifically addressed in 7 eTSA [4, 22, 24, 35, 40, 44, 62] studies and 18 mTCA studies [2, 6, 7, 17, 19, 22, 25, 37, 47, 55, 57, 60, 64, 67, 68, 70, 75, 76]. Unlike TSM, the overall fixed incidence of GTR was significantly lower in eTSA (incidence = 70.9%; 95% CI = 60.3–79.9%, p-heterogeneity = 0.45, I^2^ = 0%, 86 patients) compared to mTCA (88.5%; 95% CI = 85.9–90.7%, p-heterogeneity = 0.06, I^2^:36.5%, 786 patients) (p-interaction < 0.01; Fig. 2). In meta-regression, only higher age was associated with lower GTR in resected OGM with the eTSA approach with borderline significance (*p* = 0.05). Begg’s test for publication bias was non-significant (*p* = 0.48) (Table 4).

## Visual improvement

Visual outcomes were reported in 12 studies for eTSA [8, 12, 16, 23, 29, 30, 35, 40, 43, 61, 62, 79] and 28 studies for mTCA [3, 5, 13, 15, 21, 23, 25, 29, 32, 34, 36, 47–51, 56, 63, 65, 66, 68, 69, 72, 73, 77, 81, 82] with a total of 1139 patients presenting with visual problems [3, 5, 8, 12, 13, 15, 16, 21, 23, 25, 29, 30, 32, 34–36, 40, 43, 47–51, 53, 56, 61–63, 65, 66, 68, 69, 72, 73, 77, 79, 81, 82]. Postoperative visual improvement was significantly higher for eTSA (incidence = 77.7%; 95% CI = 70.3–83.7%, p-heterogeneity = 0.37, I^2^ = 7.90%, 167 patients) than mTCA (incidence = 60.7%; 95% CI = 57.3–64.0, p-heterogeneity < 0.01, I^2^ = 77.4%, 1139 patients) in fixed-effect models (p-interaction < 0.01). Because age and male percentage were not provided for this subgroup of patients who presented with visual problems, only continent could be assessed as a source of heterogeneity, which was not a significant source of heterogeneity for TSM resection using eTSA or MTCA. Begg’s test for publication bias was non-significant (*p* = 0.14) (Table 3). One study specifically addressed visual improvement per approach in TSM resection, finding that eTSA was associated with more visual acuity improvement (≥5%; *p*-value: 0.01), but not with improvement of visual field deficits (p-value = 0.61) [41].

Visual improvement in OGM patients was described four eTSA studies [4, 40, 44, 62] and nine mTCA studies [6, 7, 47, 57, 60, 68, 70, 75, 78] with 224 patients presenting with visual symptoms. The resulting fixed overall improvement rate was 64.5% (95% CI: 37.9–84.4%, p-heterogeneity = 0.03; I^2^ = 65.5%) for eTSA compared to 50.6% (95% CI = 42.9–58.4%, p-heterogeneity <0.01, I^2^ = 68.6%) for mTCA; however, this difference was not significant (p-interaction value: 0.33). Continent was not identified as a significant source of heterogeneity for eTSA (*p* = 0.34) and mTCA (*p* = 0.57). Begg’s test for publication bias was non-significant (*p* = 0.25) (Table 4).

## Cerebrospinal fluid leakage

**Fig. 3:**
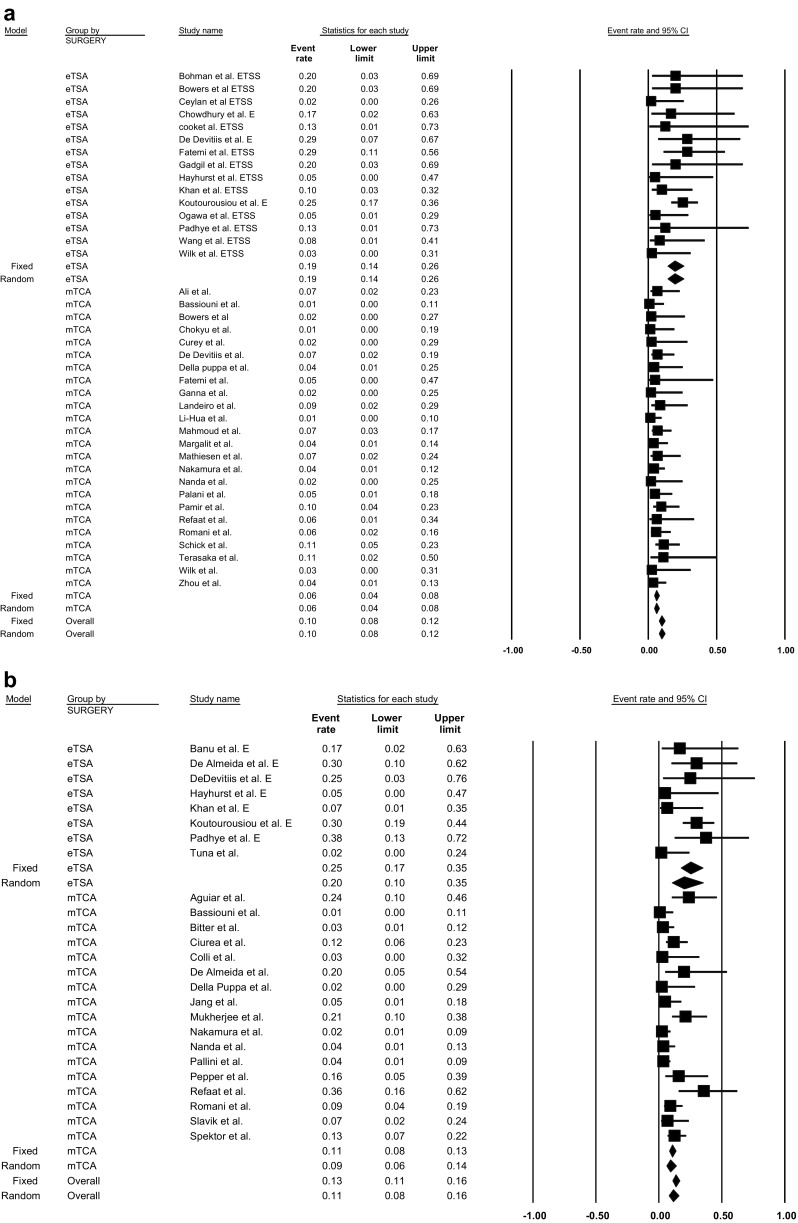
**a** Pooled prevalence of cerebrospinal fluid leak by approach for tuberculum sellae meningioma resection: endoscopic transsphenoidal approach vs. microscopic transcranial approach. P-interaction value < 0.01. CSF, cerebrospinal fluid; eTSA, endoscopic transsphenoidal approach; mTCA, microscopic transcranial approach. **b** Pooled prevalence rates of cerebrospinal fluid leak by approach for olfactory groove meningioma resection: endoscopic transsphenoidal approach vs. microscopic transcranial approach. P-interaction value < 0.01; CSF, cerebrospinal fluid; eTSA, endoscopic transsphenoidal approach; mTCA, microscopic transcranial approach

CSF leak occurrence after TSM resection was extracted from 15 eTSA studies [8, 11, 16, 20, 23, 29, 30, 35, 40, 43, 61, 62, 79, 81] and 24 mTCA studies. The overall incidence of postoperative CSF leakage was significantly higher in patients treated with the eTSA approach (incidence = 19.3%; 95% CI = 14.1–25.8%, p-heterogeneity = 0.50, I^2^ = 0%, 225 patients) than with mTSA (incidence = 5.81%; 95% CI = 4.33–7.75%, p-heterogeneity = 0.93, I^2^ = 0%, 879 patients) in fixed models (p-interaction value <0.01, Fig. 3a). Age, gender, and continent were not identified as sources of heterogeneity using meta-regression (all *p*-value > 0.05). Begg’s test revealed no significant publication bias (*p* = 0.98) (Table 3).

In OGM, 7 eTSA studies [4, 22, 24, 35, 40, 44, 62] and 17 mTCA studies [2, 6, 7, 17, 19, 22, 25, 37, 55, 57, 60, 64, 67, 68, 70, 75, 76, 78] including 889 patients described whether patients developed a CSF leak postoperatively. The overall incidence in fixed models was statistically significantly higher (p-interaction < 0.01) for eTSA (incidence = 25.1%; 95% CI = 17.5–34.8%, p-heterogeneity = 0.22, I^2^ = 25.8%) than mTCA (incidence = 10.5%; 95% CI = 8.22–13.4%, p-heterogeneity <0.01, I^2^ = 60.2%) (Fig. 3b). In meta-regression, only older age was significantly associated with a lower CSF leakage rate for mTCA (*p* < 0.01). For eTSA, age, gender, and continent were not identified as potential effect modifiers (p-interaction for all > 0.05). Begg’s test indicated no significant publication bias (*p* = 0.30) (Table 4).

## Intraoperative arterial injury

**Fig. 4:**
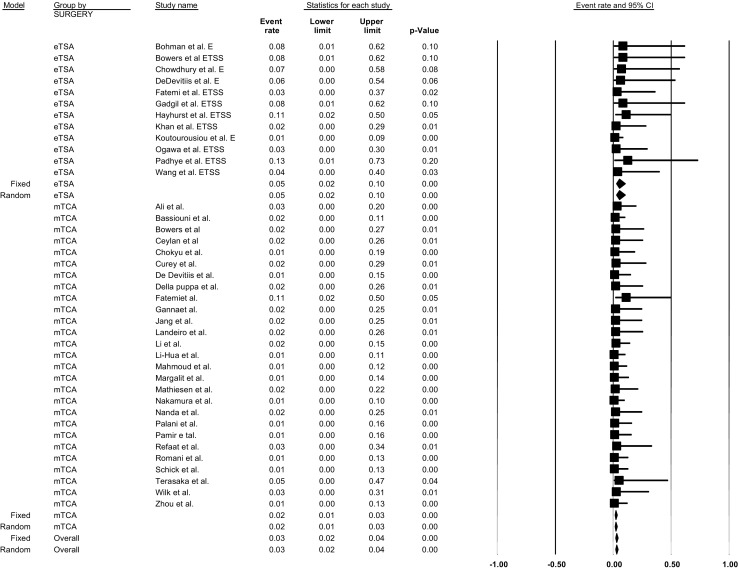
Pooled prevalence rates of intraoperative arterial injury by approach for tuberculum sellae meningioma resection: endoscopic transsphenoidal approach vs. microscopic transcranial approach. P-interaction value: 0.03. eTSA, endoscopic transsphenoidal approach; mTCA, microscopic transcranial approach

For intraoperative arterial injury, outcomes were extracted from 12 eTSA studies [8, 11, 16, 23, 29, 30, 35, 40, 43, 61, 62, 79] and 27 mTCA studies for TSM [3, 5, 11, 12, 15, 21, 23, 25, 29, 32, 36, 45, 48, 49, 51, 52, 56, 58, 63, 65, 68, 69, 72, 77, 81, 82]. The overall incidence of intraoperative arterial injury was significantly higher for eTSA (incidence = 4.89%; 95% CI = 2.33–9.94%, p-heterogeneity = 0.97, I^2^ = 0%, 225 patients) than for MTCA (incidence = 1.86%; 95% CI = 1.13–3.05%, p-heterogeneity = 0.99, I^2^ = 0%, 225 patients) in fixed effect models (p-interaction value = 0.03; Fig. 4). Trial-level covariates such as age, continent, and gender did not significantly contribute to any heterogeneity in the models for both eTSA and mTCA (all p-interaction values > 0.05). There was a significant publication bias, indicating that study results with higher arterial injury incidence tended not to be published (Begg’s test *p*-value < 0.01) (Table 3). However, the imputed overall incidence estimate for TSM was not materially different from the original incidence rate (not shown).

For OGM, the incidence of intraoperative arterial injury was extracted from 858 patients in 7 eTSA studies [4, 22, 24, 35, 44, 62] and 17 mTCA studies [2, 6, 7, 17, 19, 22, 25, 37, 55, 57, 60, 64, 67, 68, 70, 75, 76, 78]. For eTSA, the fixed overall incidence of intraoperative arterial injury was 3.88% (95% CI = 1.55–9.43%, p-heterogeneity = 0.98, I^2^ = 0%). Although lower, the incidence for mTCA was 1.62% (95% CI = 0.87–2.98%, p-heterogeneity = 0.99, I^2^ = 0%) but not significantly different (p-interaction = 0.12). Covariates such as age, gender, and continent were not identified as sources of heterogeneity for both eTSa and mTCA procedures (all p-interaction > 0.05). Although Begg’s test for publication bias indicated the presence of publication bias (*p*-value < 0.01), Egger’s test did not (*p*-value = 0.50) (Table 4). Moreover, the imputed overall incidence estimates for OGM were not materially different from the original incidence values (not shown).

## Mortality

Mortality after TSM surgery was described in a total of 10 eTSA studies [8, 11, 23, 29, 40, 43, 61, 62, 79] and 30 mTCA studies [3, 5, 11–13, 15, 21, 23, 25, 29, 30, 35, 36, 45, 48, 49, 51–53, 56, 58, 63, 65, 68, 69, 72, 77, 81, 82]. eTSA resulted in a 30-day mortality incidence of 5.15% (95% CI = 2.39–10.8, p-heterogeneity = 0.85, I^2^ = 0%, 194 patients), which was not significantly different from mTCA (incidence = 2.67%; 95% CI = 1.77–4.02, p-heterogeneity = 0.99, I^2^ = 0%, 962 patients) in fixed models (p-interaction = 0.14). Age, gender, and continent did not appear to have different incidence values based on the meta-regression results for both eTSA and mTCA (all *p* > 0.05). Begg’s test p-value for publication bias was significant, indicating that articles with higher mortality rates tend not to be published (*p* < 0.01) (Table 3); however, the trim-and-fill method suggested that the imputed overall incidence estimates for TSM were not materially different from the original incidence values (not shown).

For OGM, 7 eTSA studies [4, 22, 24, 35, 40, 44, 62] and 19 mTCA studies [2, 6, 7, 17, 19, 22, 25, 37, 47, 55, 57, 60, 64, 67, 68, 70, 75, 76, 78] including described mortality incidence. For eTSA, the overall 30-day mortality incidence was 4.27% (95% CI = 1.50–11.6%, p-heterogeneity = 0.94; I^2^ = 0%; 82 patients), which was not significantly different from the mortality incidence in the mTCA group (incidence = 3.92%, 95% CI = 2.66–5.75, p-heterogeneity = 0.74, I^2^ = 0%; 779 patients) in fixed models (p-interaction = 0.88). In a meta-regression for gender, it was identified that studies with a lower male percentage were significantly associated with a higher mortality incidence for mTCA (*p* = 0.02) but not for eTSA (*p* = 0.34), while age and continent were not. Begg’s test for publication bias was non-significant (*p* = 0.21) (Table 4).

## Random-effect models

For all the above-mentioned results, the random-effect models yielded similar results (Tables 3 and 4).

## Blood loss, operating time, and length of stay in hospital

For blood loss, operating time, and length of hospital stay, a quantitative meta-analysis was not feasible because of the paucity of studies reporting them; hence, these few studies were systematically reviewed. In TSM, mean blood loss ranged from 448 to 970 ml in three studies describing mTCA compared to 200 to 617 ml for eTSA [21, 30, 41, 47]. The mean operating time ranged from 375 to 444 min for eTSA in two studies and from 116 to 426 min for mTCA in four studies [21, 23, 41, 47, 69]. Hospital length of stay ranged from 6 to 21 days in one study in patients treated by an eTSA [23].

For OGM, blood loss was only reported in one case series in patients operated with an interhemispheric approach (mean: 570.9 ml, SD: 442) [47]. The mean hospital length of stay for eTSA ranged from 11 to 13.5 days in two studies [9, 13] compared to 8.5 to 18 days for mTCA [7, 22, 24, 78]. Of these studies, one described the mean length of stay in both approaches, with a mean length of stay of 11 days for eTSA compared to 8.5 days in mTCA (*p* = 0.54) [22]. Operating time ranged from 6 to 10 h in one study reporting outcomes from eTSA [24]. In a study examining patients with an interhemispheric approach, the mean operating time was 209 min (standard deviation: 103) [47].

## Discussion

In this meta-analysis, eTSA was not shown to be superior to mTCA for resection of both OGMs and TSMs. Only in patients with preoperative visual deficits due to TSM, eTSA seems superior to mTCA, but with great hetereogeneity. In patients with TSM, eTSA resulted in higher rates of visual improvement, similar rates of GTR, and more CSF leaks and intraoperative arterial injury, while in patients with OGM, results of both techniques were similar for visual improvement and intraoperative arterial injury, but worse in patients operated with eTSA for GTR and CSF leaks. There seems to be no substantial difference in perioperative blood loss, operating time, or length of hospital stay between the two approaches. There was no substantial difference between incidence rates in the fixed- and random-effect models. This could be explained by a relative lack of difference between the study populations in the studies, which could have been implicated in the case of a difference between the models. However, mTCA was associated with considerable heterogeneity for outcomes visual improvement in TSMs and CSF leak for OGMs, which could reflect a relatively greater inter-study variability for these outcomes.

Although no significant difference was identified in GTR rate for TSM, mTCA resulted in higher GTR rates in OGM. As OGMs are located more anteriorly than TSM, an extended eTSA approach is needed for OGM, which requires more extensive drilling of the anterior skull base and a potential suboptimal view because of the angle of the scope. However, it should also be noted that GTR was not always the primary the goal of surgery (e.g., the goal could be preserving vision) [43, 72]. Furthermore, many other factors seem to influence GTR rate. One factor may be the learning curve associated with eTSA, as seen with pituitary adenoma resection [10, 14, 46]. Also, tumor factors such as large size and vascular enhancement can significantly lower the GTR rate for eTSA, as seen in one study in TSM [43]. Furthermore, presence of a “cortical cuff” (a layer of brain between the tumor capsule and cerebral vessels) on MRI was associated with more GTR in OGM [40].

For visual improvement, it remains to be determined whether eTSA is truly associated with more visual improvement than mTCA in TSM as correction for the heterogeneity among mTCA studies could not be done. Therefore, the difference witnessed may be insignificant as seen with OGM. Furthermore, as the variance in reporting of tumor size did not allow for it to be incorporated in a meta-regression, the TSMs in the eTSA group may be smaller compared to the mTCA group. However, regarding visual outcomes, one study looking at the mTCA approach suggests that visual outcomes are associated with age and duration of visual symptoms but not with actual tumor size [28].

For both OGM and TSM, eTSA was significantly associated with more CSF leakage. However, prophylactic lumbar drain placement varied greatly; in some studies, almost all patients were given a prophylactic preoperative lumbar drain, while in other studies none of the included patients were drained [8, 24, 30, 35, 40, 44]. Also, the different studies used different reconstruction techniques (e.g., introduction of a vascularized flap and use of certain glues), although this caused no considerable heterogeneity among the studies [40, 44, 62]. Another factor in the postoperative CSF leakage rate may be the neurosurgeon’s level experience. Although the difference was not significant, in a small number of patients, one group had two leaks in their first group of patients (*n* = 8) compared to none in the latter group (*n* = 12) [40]. Also, use of a vascularized flap for reconstruction of the skull base seems to bring the CSF leakage rate down considerably [40, 43, 62]. Still, this rate is considerably higher than the overall incidence calculated for mTCA. Further improvement with more sophisticated reconstruction techniques following eTSA may bring the rate of CSF leakage down to those reported for mTCA.

MTCA for TSM resulted in a significantly lower rate of intraoperative arterial injury compared to eTSA. However, this seems not to have caused a significant difference in mortality. Nevertheless, the relatively low number of patients treated with an eTSA may have caused a relatively low power, as the p-interaction value for mortality for TSM approaches significance (*p* = 0.14). A significant association between intraoperative arterial injury and eTSA was not seen in OGM; again, this may be explained by low power and the small number of studies, but also because of the anterior location of the tumor.

Previously, two reviews have described a comparison between eTSA and mTCA for both TSM and OGM. The first review identified a higher GTR rate and less CSF leakage associated with mTCA for both OGM and TSM (*p* < 0.01 for both, using the chi-squared test and Fisher’s exact test, respectively), which is similar to our findings except for the GTR rate for TSM [42]. A second review found significantly more visual improvement (p < 0.01) and CSF leakage (p < 0.01) for eTSA and no difference in mortality (*p* = 0.15) for TSM and OGM together, similar to our findings. eTSA was also found to be associated with a lower GTR rate (p < 0.01) compared to mTCA, which was only the case in OGM in this meta-analysis [71]. Finally, the authors of a meta-analysis for TSM found that eTSA was significantly associated with CSF leakage (OR: 3.9; 95% CI: 1.15–15.75, *p* < 0.05) and visual improvement (OR 1.5; 95% CI 1.18, 1.82, p < 0.05), which again is similar to our results [18].

Strengths of this study include an extensive review of the literature and evaluation of outcomes such as arterial injury, length of hospital stay, and blood loss. The use of both fixed- and random-effect models, evaluation of heterogeneity between the included studies, and assessment of publication bias ensures a rigorous evaluation of outcomes with appropriate valuation of the results. All outcomes were also subjected to meta-regression for various study characteristics where possible to try to identify sources of heterogeneity between the studies.

There are several limitations of this meta-analysis. First, the decision to discard studies published before 2004 produces a limitation. The decision to do so was based on the assumption that also mTCA outcomes improve over time with continual innovation and that meningiomas were not reported to be resected with an eTSA before that time [26, 38]. Regarding the included studies, only case series were identified, resulting in the inability of calculation overall odds ratios. There is probably also a great difference between the population of patients who were deemed eligible for an eTSA resection compared to those resected with mTCA because of the size, extension, and invasion of the tumors (confounding by indication). Furthermore, one could argue that only looking at perioperative outcomes may not be conclusive as especially recurrence happens during follow-up. However, as the GTR and World Health Organization (WHO) grade remain the main prognostic factors for predicting recurrence, opting for eTSA should be done with great caution as high-grade meningiomas may be harder to resect completely [59, 74]. However, it was not possible to correct for meningioma size, which is unfortunate as very small meningiomas may show very different results. Furthermore, it was not possible to correct for WHO grade, which could theoretically alter the results [31]. Also, the choice of approach varied greatly among mTCA approach studies [1, 2, 5–7, 9, 47, 51, 56, 64, 70].

Indications for eTSA vary between groups. One group reported operating on all midline meningiomas regardless of size, extension, or configuration except for those tumors that extend from the anterior clinoid process [43]. It has also been suggested that if the tumor extends laterally over the internal carotid artery, chances of GTR are limited [61]. Others have suggested that larger tumors, tumors that extend laterally, involve vasculature, or are calcified are also lesser candidates [23, 44]. Therefore, confounding by indication cannot be ruled out, especially since the patients in these studies were not randomized to either treatment. As a result, the exact indications and contraindications for eTSA remain to be determined.

Future studies should, therefore, focus on identifying clear indications for eTSA for OGM and TSM and its safety by direct comparison in a randomized study. Such a study should ideally be conducted in a research setting by experienced surgeons, as its safety has not been prospectively compared to mTCA and as both approaches seem to come with a considerable learning curve, which results in different outcomes [43]. Given the observation that younger patients seem to benefit more from eTSA compared to older patients (*p* = 0.02, *n* = 34), it is not unlikely that specific groups might benefit more from one of the approaches [39]. Probably, patients with relatively small (<3 cm) midline TSMs would probably be the best early candidates. These patients may benefit from a potential higher incidence of visual improvement postoperatively and the relative invasiveness of the eTSA approach. Further evaluation could be focused on characteristics such as size, a cortical cuff, and WHO grading to identify the best potential candidates for either approach [40]. However, due to the low incidence of TSMs and OGMs in general and the great variety in anatomical characteristics among them, this may be challenging. Therefore, other trial designs—e.g., a registry—should be considered when answering this question. Also, future improvement of the instruments used (e.g., 3D endoscopes or glues) may improve the results obtained by eTSA over time [33].

## Conclusion

This meta-analysis indicates that the endoscopic transsphenoidal approach (eTSA) has not been shown to be superior to the microscopic transsphenoidal approach (mTCA) for either olfactory groove meningiomas (OGMs) or tuberculum sellae menigniomas (TSMs). More specifically, eTSA was associated with lower GTR rate for OGMs and higher rate of arterial injury for TSMs compared to mTCA. Furthermore, eTSA was associated with more CSF leaks in both OGMs and TSMs compared to mTCA. On the other hand, eTSA was associated with a higher rate of visual improvement postoperatively compared to mTCA in TSMs, which was not observed for OGMs. All conclusions should, however, be interpreted with caution because of the limitations of this study.

## Electronic supplementary material

Supplementary Table 1 Search syntax (DOCX 12 kb)

## References

[1] Abbassy M, Woodard TD, Sindwani R, Recinos PF (2016). An overview of anterior skull base meningiomas and the endoscopic endonasal approach. Otolaryngol Clin N Am.

[2] Aguiar PH, Tahara A, Almeida AN, Simm R, Silva AN, Maldaun MV, Panagopoulos AT, Zicarelli CA, Silva PG (2009). Olfactory groove meningiomas: approaches and complications. J Clini Neurosci.

[3] Ali MZ, El-Mekawi S, Al-Azzazi A (2010). Tuberculum sellae meningiomas: surgical results and outcome in 30 cases. Egyptian J Neurol, Psychiatry Neurosurg.

[4] Banu MA, Mehta A, Ottenhausen M, Fraser JF, Patel KS, Szentirmai O, Anand VK, Tsiouris AJ, Schwartz TH (2016). Endoscope-assisted endonasal versus supraorbital keyhole resection of olfactory groove meningiomas: comparison and combination of 2 minimally invasive approaches. J Neurosurg.

[5] Bassiouni H, Asgari S, Stolke D (2006). Tuberculum sellae meningiomas: functional outcome in a consecutive series treated microsurgically. Surg Neurol.

[6] Bassiouni H, Asgari S, Stolke D (2007). Olfactory groove meningiomas: functional outcome in a series treated microsurgically. Acta Neurochir.

[7] Bitter AD, Stavrinou LC, Ntoulias G, Petridis AK, Dukagjin M, Scholz M, Hassler W (2013). The role of the pterional approach in the surgical treatment of olfactory groove meningiomas: a 20-year experience. J Neurol Surg. Part B, Skull base.

[8] Bohman LE, Stein S, Newman JG, Palmer J, Adappa N, Khan A, Sitterley TT, Chang D, Lee JY (2012) Decision analysis: endoscopic versus open resection of tuberculum sellae meningiomas. J Neurol Surg, Part B: Skull Base 73 10.1159/000343794 23107968

[9] Bohman LE, Stein SC, Newman JG, Palmer JN, Adappa ND, Khan A, Sitterley TT, Chang D, Lee JY (2012). Endoscopic versus open resection of tuberculum sellae meningiomas: a decision analysis. ORL J Otorhinolaryngol Relat Spec.

[10] Bokhari AR, Davies MA, Diamond T (2013). Endoscopic transsphenoidal pituitary surgery: a single surgeon experience and the learning curve. Br J Neurosurg.

[11] Bowers CA, Altay T, Couldwell WT (2011). Surgical decision-making strategies in tuberculum sellae meningioma resection. Neurosurg Focus.

[12] Ceylan S, Anik I, Koc K, Cabuk B (2015). Extended endoscopic transsphenoidal approach infrachiasmatic corridor. Neurosurg Rev.

[13] Chen LH, Chen L, Liu LX (2011). Microsurgical management of tuberculum sellae meningiomas by the frontolateral approach: surgical technique and visual outcome. Clin Neurol Neurosurg.

[14] Chi F, Wang Y, Lin Y, Ge J, Qiu Y, Guo L (2013). A learning curve of endoscopic transsphenoidal surgery for pituitary adenoma. J Craniofac Surg.

[15] Chokyu I, Goto T, Ishibashi K, Nagata T, Ohata K (2011). Bilateral subfrontal approach for tuberculum sellae meningiomas in long-term postoperative visual outcome: clinical article. J Neurosurg.

[16] Chowdhury FH, Haque MR, Goel AH, Kawsar KA (2012). Endoscopic endonasal extended transsphenoidal removal of tuberculum sellae meningioma (TSM): an experience of six cases. Br J Neurosurg.

[17] Ciurea AV, Iencean SM, Rizea RE, Brehar FM (2012). Olfactory groove meningiomas: a retrospective study on 59 surgical cases. Neurosurg Rev.

[18] Clark AJ, Jahangiri A, Garcia RM, George JR, Sughrue ME, McDermott MW, El-Sayed IH, Aghi MK (2013). Endoscopic surgery for tuberculum sellae meningiomas: a systematic review and meta-analysis. Neurosurg Rev.

[19] Colli BO, Carlotti CG, Assirati JA, Santos MB, Neder L, Santos AC, Batagini NC (2007). Olfactory groove meningiomas: surgical technique and follow-up review. Arq Neuropsiquiatr.

[20] Cook SW, Smith Z, Kelly DF (2004). Endonasal transsphenoidal removal of tuberculum sellae meningiomas: technical note. Neurosurgery.

[21] Curey S, Derrey S, Hannequin P, Hannequin D, Freger P, Muraine M, Castel H, Proust F (2012). Validation of the superior interhemispheric approach for tuberculum sellae meningioma: clinical article. J Neurosurg.

[22] de Almeida JR, Carvalho F, Vaz Guimaraes Filho F, Kiehl TR, Koutourousiou M, Su S, Vescan AD, Witterick IJ, Zadeh G, Wang EW, Fernandez-Miranda JC, Gardner PA, Gentili F, Snyderman CH (2015). Comparison of endoscopic endonasal and bifrontal craniotomy approaches for olfactory groove meningiomas: a matched pair analysis of outcomes and frontal lobe changes on MRI. J Clin Neurosci.

[23] De Divitiis E, Esposito F, Cappabianca P, Cavallo LM, De Divitiis O (2008). Tuberculum sellae meningiomas: high route or low route? A series of 51 consecutive cases. Neurosurgery.

[24] de Divitiis E, Esposito F, Cappabianca P, Cavallo LM, de Divitiis O, Esposito I (2008). Endoscopic transnasal resection of anterior cranial fossa meningiomas. Neurosurg Focus.

[25] Della Puppa A, D’Avella E, Rossetto M, Volpin F, Rustemi O, Gioffrè G, Lombardi G, Rolma G, Scienza R (2015). Open transcranial resection of small (<35 mm) meningiomas of the anterior midline skull base in current microsurgical practice. World Neurosurg.

[26] DeMonte F, McDermott MW, Al-Mefty A (2011). Al-Mefty’s Meningiomas.

[27] DerSimonian R, Laird N (1986). Meta-analysis in clinical trials. Control Clin Trials.

[28] Fahlbusch R, Schott W (2002). Pterional surgery of meningiomas of the tuberculum sellae and planum sphenoidale: surgical results with special consideration of ophthalmological and endocrinological outcomes. J Neurosurg.

[29] Fatemi N, Dusick JR, de Paiva Neto MA, Malkasian D, Kelly DF (2009). Endonasal versus supraorbital keyhole removal of craniopharyngiomas and tuberculum sellae meningiomas. Neurosurgery.

[30] Gadgil N, Thomas JG, Takashima M, Yoshor D (2013). Endoscopic resection of tuberculum sellae meningiomas.. J Neurol Surg Part B Skull Base.

[31] Gallagher MJ, Jenkinson MD, Brodbelt AR, Mills SJ, Chavredakis E (2016). WHO grade 1 meningioma recurrence: are location and Simpson grade still relevant?. Clin Neurol Neurosurg.

[32] Ganna A, Dehdashti AR, Karabatsou K, Gentili F (2009). Fronto-basal interhemispheric approach for tuberculum sellae meningiomas; long-term visual outcome. Br J Neurosurg.

[33] Gerlach R, Meyer A, Kellner G (2014) Comparison of 2D HD and 3D endoscopy during surgery for perisellar pathologies. Exp Clin Endocrinol Diabetes 122

[34] Goel A, Muzumdar D (2005). Surgical strategy for tuberculum sellae meningiomas. Neurosurg Q.

[35] Hayhurst C, Sughrue ME, Gore PA, Bonney PA, Burks JD, Teo C (2016). Results with expanded endonasal resection of skull base meningiomas: technical nuances and approach selection based on an early experience. Turkish Neurosurg.

[36] Jang WY, Jung S, Jung TY, Moon KS, Kim IY (2012). The contralateral subfrontal approach can simplify surgery and provide favorable visual outcome in tuberculum sellae meningiomas. Neurosurg Rev.

[37] Jang WY, Jung S, Jung TY, Moon KS, Kim IY (2013). Preservation of olfaction in surgery of olfactory groove meningiomas. Clin Neurol Neurosurg.

[38] Jho HD, Ha HG (2004). Endoscopic endonasal skull base surgery: part 1—the midline anterior fossa skull base. Minim Invasive Neurosurg.

[39] Jones SH, Iannone AF, Patel KS, Anchouche K, Raza SM, Anand VK, Schwartz TH (2016). The impact of age on long-term quality of life after endonasal endoscopic resection of skull base meningiomas. Neurosurgery.

[40] Khan OH, Anand VK, Schwartz TH (2014). Endoscopic endonasal resection of skull base meningiomas: the significance of a "cortical cuff" and brain edema compared with careful case selection and surgical experience in predicting morbidity and extent of resection. Neurosurg Focus.

[41] Kitano M, Taneda M, Nakao Y (2007). Postoperative improvement in visual function in patients with tuberculum sellae meningiomas: results of the extended transsphenoidal and transcranial approaches. J Neurosurg.

[42] Komotar RJ, Starke RM, Raper DM, Anand VK, Schwartz TH (2012). Endoscopic endonasal versus open transcranial resection of anterior midline skull base meningiomas. World Neurosurg.

[43] Koutourousiou M, Fernandez-Miranda JC, Stefko ST, Wang EW, Snyderman CH, Gardner PA (2014). Endoscopic endonasal surgery for suprasellar meningiomas: experience with 75 patients: clinical article. J Neurosurg.

[44] Koutourousiou M, Fernandez-Miranda JC, Wang EW, Snyderman CH, Gardner PA (2014). Endoscopic endonasal surgery for olfactory groove meningiomas: outcomes and limitations in 50 patients. Neurosurg Focus.

[45] Landeiro JA, Gonçalves MB, Guimarães RD, Klescoski J, Correa JLA, Lapenta MA, Máia O (2010). Tuberculum sellae meningiomas: surgical considerations. Arq Neuropsiquiatr.

[46] Leach P, Abou-Zeid AH, Kearney T, Davis J, Trainer PJ, Gnanalingham KK (2010). Endoscopic transsphenoidal pituitary surgery: evidence of an operative learning curve. Neurosurgery.

[47] Leveque S, Derrey S, Martinaud O, Gerardin E, Langlois O, Freger P, Hannequin D, Castel H, Proust F (2011). Superior interhemispheric approach for midline meningioma from the anterior cranial base. Neuro-Chirurgie.

[48] Li X, Liu M, Liu Y, Zhu S (2007). Surgical management of tuberculum sellae meningiomas. J Clin Neurosci.

[49] Li-Hua C, Ling C, Li-Xu L (2011). Microsurgical management of tuberculum sellae meningiomas by the frontolateral approach: surgical technique and visual outcome. Clin Neurol Neurosurg.

[50] Liu HC, Qiu E, Zhang JL, Kang J, Li Y, Li Y, Jiang LB, Fu JD (2015). Surgical indications of exploring optic canal and visual prognostic factors in neurosurgical treatment of tuberculum sellae meningiomas. Chin Med J.

[51] Mahmoud M, Nader R, Al-Mefty O (2010). Optic canal involvement in tuberculum sellae meningiomas: influence on approach, recurrence, and visual recovery. Neurosurgery.

[52] Margalit N, Shahar T, Barkay G, Gonen L, Nossek E, Rozovski U, Kesler A (2013). Tuberculum sellae meningiomas: surgical technique, visual outcome, and prognostic factors in 51 cases. J Neurol Surg, Part B: Skull Base.

[53] Mathiesen T, Kihlström L (2006). Visual outcome of tuberculum sellae meningiomas after extradural optic nerve decompression. Neurosurgery.

[54] Moher D, Liberati A, Tetzlaff J, Altman DG, Group P (2010). Preferred reporting items for systematic reviews and meta-analyses: the PRISMA statement. Int J Surg.

[55] Mukherjee S, Thakur B, Corns R, Connor S, Bhangoo R, Ashkan K, Gullan R (2015). Resection of olfactory groove meningioma—a review of complications and prognostic factors. Br J Neurosurg.

[56] Nakamura M, Roser F, Struck M, Vorkapic P, Samii M (2006). Tuberculum sellae meningiomas: clinical outcome considering different surgical approaches. Neurosurgery.

[57] Nakamura M, Struck M, Roser F, Vorkapic P, Samii M (2008). Olfactory groove meningiomas: clinical outcome and recurrence rates after tumor removal through the frontolateral and bifrontal approach. Neurosurgery.

[58] Nanda A, Ambekar S, Javalkar V, Sharma M (2013). Technical nuances in the management of tuberculum sellae and diaphragma sellae meningiomas. Neurosurg Focus.

[59] Nanda A, Bir SC, Maiti TK, Konar SK, Missios S, Guthikonda B (2017) Relevance of Simpson grading system and recurrence-free survival after surgery for World Health Organization grade I meningioma. J Neurosurg 126: 201-2011 10.3171/2016.1.JNS151842 27058201

[60] Nanda A, Maiti TK, Bir SC, Konar SK, Guthikonda B (2016). Olfactory groove meningiomas: comparison of extent of frontal lobe changes after lateral and bifrontal approaches. World Neurosurg.

[61] Ogawa Y, Tominaga T (2012). Extended transsphenoidal approach for tuberculum sellae meningioma—what are the optimum and critical indications?. Acta Neurochir.

[62] Padhye V, Naidoo Y, Alexander H, Floreani S, Robinson S, Santoreneos S, Wickremesekera A, Brophy B, Harding M, Vrodos N, Wormald PJ (2012). Endoscopic endonasal resection of anterior skull base meningiomas. Otolaryngol-Head Neck Surg (US).

[63] Palani A, Panigrahi MK, Purohit AK (2012). Tuberculum sellae meningiomas: a series of 41 cases; surgical and ophthalmological outcomes with proposal of a new prognostic scoring system. J Neurosci Rural Pract.

[64] Pallini R, Fernandez E, Lauretti L, Doglietto F, D’Alessandris QG, Montano N, Capo G, Meglio M, Maira G (2015) Olfactory groove meningioma: report of 99 cases surgically treated at the Catholic University School of Medicine, Rome. World Neurosurg 83:219–231.e211–213 10.1016/j.wneu.2014.11.001 25464274

[65] Pamir MN, Ozduman K, Belirgen M, Kilic T, Ozek MM (2005). Outcome determinants of pterional surgery for tuberculum sellae meningiomas. Acta Neurochir.

[66] Park CK, Jung HW, Yang SY, Seol HJ, Paek SH, Kim DG (2006). Surgically treated tuberculum sellae and diaphragm sellae meningiomas: the importance of short-term visual outcome. Neurosurgery.

[67] Pepper JP, Hecht SL, Gebarski SS, Lin EM, Sullivan SE, Marentette LJ (2011). Olfactory groove meningioma: discussion of clinical presentation and surgical outcomes following excision via the subcranial approach. Laryngoscope.

[68] Refaat MI, Eissa EM, Ali MH (2015). Surgical management of midline anterior skull base meningiomas: experience of 30 cases. Turkish Neurosurg.

[69] Romani R, Laakso A, Kangasniemi M, Niemela M, Hernesniemi J (2012). Lateral supraorbital approach applied to tuberculum sellae meningiomas: experience with 52 consecutive patients. Neurosurgery.

[70] Romani R, Lehecka M, Gaal E, Toninelli S, Celik O, Niemela M, Porras M, Jaaskelainen J, Hernesniemi J (2009). Lateral supraorbital approach applied to olfactory groove meningiomas: experience with 66 consecutive patients. Neurosurgery.

[71] Ruggeri AG, Cappelletti M, Fazzolari B, Marotta N, Delfini R (2016). Frontobasal midline meningiomas: is it right to shed doubt on the transcranial approaches? Updates and review of the literature. World Neurosurg.

[72] Schick U, Hassler W (2005). Surgical management of tuberculum sellae meningiomas: involvement of the optic canal and visual outcome. J Neurol Neurosurg Psychiatry.

[73] Seol HJ, Park HY, Nam DH, Kong DS, Lee JI, Kim JH, Park K (2013). Clinical outcomes of tuberculum sellae meningiomas focusing on reversibility of postoperative visual function. Acta Neurochir.

[74] Simpson D (1957). The recurrence of intracranial meningiomas after surgical treatment. J Neurol Neurosurg Psychiatry.

[75] Slavik E, Radulovic D, Tasic G (2007). Olfactory groove meningiomas. Acta Chirurgica Iugoslavica.

[76] Spektor S, Valarezo J, Fliss DM, Gil Z, Cohen J, Goldman J, Umansky F (2005). Olfactory groove meningiomas from neurosurgical and ear, nose, and throat perspectives: approaches, techniques, and outcomes. Neurosurgery.

[77] Terasaka S, Asaoka K, Kobayashi H, Yamaguchi S (2011). Anterior interhemispheric approach for tuberculum sellae meningioma. Neurosurgery.

[78] Tuna H, Bozkurt M, Ayten M, Erdogan A, Deda H (2005). Olfactory groove meningiomas. J Clin Neurosci.

[79] Wang Q, Lu XJ, Ji WY, Yan ZC, Xu J, Ding YS, Zhang J (2010). Visual outcome after extended endoscopic endonasal transsphenoidal surgery for tuberculum sellae meningiomas. World Neurosurg.

[80] Wells G, Shea B, O’Connell D, Peterson J, Welch V, Losos M, Tugwell P The Newcastle-Ottawa scale (NOS) for assessing the quality of nonrandomised studies in meta-analyses. http://www.medicine.mcgill.ca/rtamblyn/Readings/The Newcastle Scale for assessing the quality of nonrandomised studies in meta-analyses.pdf. Accessed 13 Oct 2016

[81] Wilk A, G ZI, Witek P, Koziarski A (2015) Outcome assessment after surgical treatment of tuberculum sellae meningiomas—a preliminary report. Turkish Neurosurg 26:824–832 10.5137/1019-5149.JTN.14160-15.1 27560535

[82] Zhou H, Wu Z, Wang L, Zhang J (2016). Microsurgical treatment of tuberculum sellae meningiomas with visual impairments: a Chinese experience of 56 cases. Turkish Neurosurg.

