# Supplementary material for: The endoscopic endonasal approach is not superior to the microscopic transcranial approach for anterior skull base meningiomas—a meta-analysis
Source: Acta Neurochir (Wien). 2017 Nov 10;160(1):59–75. doi: 10.1007/s00701-017-3390-y (PMC5735207; doi:10.1007/s00701-017-3390-y)
Supplement: Supplementary file 1 — Search syntax (DOCX 12 kb) [file 701_2017_3390_MOESM1_ESM.docx]

Supplementary Table 1: Search syntax

|  |
| --- |
| Pubmed (09-12-2016) from 2004 |
| (Tuberculum[Title/Abstract] OR Suprasellar[Title/Abstract] OR sellar[Title/Abstract] OR sella[Title/Abstract] OR sellae[Title/Abstract] OR cribriform[Title/Abstract] OR Planum[Title/abstract] OR Sphenoid*[Title/abstract] OR olfactory[Title/abstract] OR sphenoid bone[MeSH Terms] OR anterior skull base[Title/Abstract] OR "Cranial Fossa, Anterior"[Mesh]) AND (Meningioma*[Title/Abstract] OR meningioma[MeSH Terms] OR meningeoma*[Title/abstract] OR meningeal neoplasms[MeSH Terms] OR TSM[Title/abstract] OR OGM[Title/abstract] OR PSM[Title/abstract]) |
| Embase (09-12-2016) from 2004 |
| (Olfactory:ab,ti OR tuberculum:ab,ti OR suprasellar:ab,ti OR sellar:ab,ti OR sella:ab,ti OR Sellae:ab,ti OR planum:ab,ti OR cribriform:ab,ti OR sphenoid*:ab,ti OR 'sphenoid'/exp OR 'anterior skull base':ab,ti) AND (meningioma*:ab,ti OR 'meningioma'/exp OR meningeoma*ab,ti OR TSM:ab,ti OR OGM:ab,ti OR PSM:ab,ti) |
